# The Prevalence of fungal infections in children with hematologic malignancy in Ali-Asghar Children Hospital between 2005 and 2010 

**Published:** 2015-03-15

**Authors:** Sh Ansari, E Shirzadi, M Elahi

**Affiliations:** 1 Assistant professor of Pediatrician, Oncologist, Ali-Asghar Children Hospital, Iran University of Medical Sciences, Tehran, Iran; 2 General practitioner, Ali-Asghar Children Hospital, Iran University of Medical Sciences, Tehran, Iran; 3 General practitioner, Ali-Asghar Children Hospital, Iran University of Medical Sciences, Tehran, Iran

**Keywords:** Candidiasis, Aspergillosis, Mucormycosis, Hematologic malignancy, Children

## Abstract

**Background:**

A fungal infection represents a growing problem in children with hematologic malignancies, during chemotherapy induced neutropenia. Fungal colonization is considered a major risk factor for subsequent fungal infections. The aim of this retrospective study was to evaluate prevalence of fungal infection among children admitted to hospital between 2005 and 2010 in Tehran, Iran.

**Materials and Methods:**

617 hematological patients in the age range of neoteric to 19 years old were enrolled and 87 cases with invasive fungal infections were extracted from patients' files and documented. Diagnosis of fungal infections was based on the local biopsy and pathology for mucormycosis, blood culture, urine culture and clinical examination for candidasis and galactomannan for aspergillus.

**Results:**

the mean age of cancer diagnosis was 6.33 years old and the mean age of fungal infection was 7.95 years old. The majority of the infections was caused by candidia spp (74.7%), followed by aspergillus spp (17.2%) and zygomycetes (11.5%). Among candidiasis patients, oral infection had the highest manifestation (92.3%) whereas in 10 of 15 patients with aspergillus, the infectious site was the lung. There was a significant association between mortality and the type of fungal infection (p <0.0001).

**Conclusion:**

Our finding suggests that there is a high rate of fungal infections in children receiving remission therapy for onco-hematology. These results help improve the management of these patients, however Further studies are needed.

## Introduction

Leukemia causes a significant decrease in the number of white blood cells as well as loss of their function, consequently a decrease in body's ability to find pathogens. Furthermore, leukemia alters other hematologic regimens, for instance hematologic malignancies are one of the four causes of children's mortality worldwide [1]. Risk factors of Leukemia include age, gender, genes, family history, radiation, etc [2]. The risk of fungal infection incidence in hematologic malignancy is dependent on the types of cytotoxic or immunosuppressive drugs that are used and patients' response [3]. The most important risk factors of fungal infections in malignancies are age, advanced underlying diseases, suppression of the autoimmune system, the myeloid suppression, colonization on the epithelium layers, environmental contacts, and physical damage to the body's preservative layers [4].

Patients with hematologic cancer who receive continuous chemotherapies are more susceptible to develop fungal infections compared to individuals with solid tumors receiving remission chemotherapies twice a month.

Candida, thrush or yeast infection is a fungal infection (mycosis) of any species from the genus candida. Candida albicans is the most common agent of candidiasis in humans where seven types of Candida are known as opportunist pathogens [5]. This microorganism is found on the skin, nail, vagina, urinary tract as a human symbion. Candida species causes local or systemic infections in favorable circumstances and is the fourth common cause of blood infection in some countries. Candidasis has increased dramatically since 1940 due to the vast number of prescriptions of antibiotics, steroids and other immunosuppressive drugs [6].

The agent aspergillosis is spread in the environment, such as the air, soil, dust, spice, coffee, milk powder, etc. The most common route of transmission of aspergillosis is inhalation, resulting in the infection of the sinuses and the lung. The macrophages and neutrophils are the most significant defense against the aspergillosis in the respiratory system. The aspergillus flavous is the pathogen of 67% of infections in malignant diseases. 

 Mucormycosis (zygomycosis) is an infection caused by fungi in mucorales, mutirlal and entomophthorales. The most common route of transmission of zygomycetes fungi is inhalation of spores from the environment. The other route is inoculating the microorganism through an injured skin [7]. Mucormycosis commonly occurs in rhinocerebral or pulmonary diseases and less frequently in gastrointestinal diseases. Clinical manifestations of invasive mucormycosis are tissue necrosis and subsequent thrombosis [7].These organisms are ubiquitous and generally saprophytic, rarely causing disease in immunocompetent hosts, but are the third most common cause of invasive fungal infections in immunocompromised patients, especially stem cell transplant recipients and patients with underlying hematologic malignancies.

 The neutropenia and chemotherapy both damage the mucosa, and the use of antibiotics over long periods causes the normal body flora to perish. Candida and aspergillus are the common fungal infections in the hematologic malignancies. These pathogens comprise 75% of fungal infections in the public, and are the causes of 25-60% of mortalities [8]. 

 Invasive fungal disease is associated with increased morbidity and mortality in hematologic malignancy patients and hematopoietic stem cell transplant recipients. Timely diagnosis and treatment of invasive fungal diseases in hematologic malignancy patients are very critical, decreasing the mortality rate. There have been several studies on the prevalence of fungal infections in adults, however very reported of such prevalence in children [9]. In this retrospective study, we assessed registered details in files of admitted leukemic children in a hospital. By timely diagnosis of fungal infections, we can prevent the spreading of fungal infections and manage these patients by prescribing prophylactic anti-fungal treatments.

## Materials and methods

All files of admitted leukemic patients between 2005 to 2010 in Ali-asghar Children Hospital, Tehran, Iran were evaluated. Any file with incorrect or imprecise data was omitted. Eighty seven patients were eligible to be included in the study. Diagnosis of fungal infections was based on the local biopsy and pathology for mucormycosis, blood culture, urine culture and clinical examination for candidasis and galactomannan for aspergillus. This was an analytical and retrospective study. Retrospective studies are fast, easy, low cost and are appropriate for certain topics with few samples similar to current study. Collected data include demographic information, type of cancer, age of cancer diagnosing, age of infection by fungal, type of microorganism which caused the infection, location of fungal infection, the phase of cancer when fungal infection was diagnosed ( induction, maintenance, relapse) and the patients' results after treatment (recovery or death). The type of microorganism was collected from the patient files based on pathology. 

 Because the sampling of this study was kind of census, all children who had hematologic malignancies between2005 and 2010 referred to the Ali-Asghar hospital were entered into the study. The study had two exclusion factors: any patient who was not afflicted with fungal infections and the patients who had an incompleted checklist. Census methods are useful for low and restricted samples. As a result, this method can subsume all the individuals into a study.

 Collected data was analyzed with the SPSS 16 program. Age data were assessed with the correlation test. The Eta test was used to evaluate the correlation between the quantitative and qualitative data. The result of Eta test is between 0 and 1 where 0-0.33 range represents no or weak correlation, 0.34-0.66 range represents intermediate correlation that requires more studies and 0.67-1 range represents strong correlation. Chi-Square test was used to evaluate the correlation between the qualitative data.

## Results

 Out of 87 eligible patients of this study, 55 were boys (63%) and 32 were girls (37%). The mean age was 8.00 years old (ranged from 1 to 19) where 42 (48.2%) were under 6 years old. In the past, individuals over the age of 16 years old had been admitted in the pediatric ward of Ali-Asghar hospital. 63 patients (72.5%) had acute lymphoblastic leukemia (ALL), 15 (17.3%) cases had acute myeloid leukemia (AML), 5 (5.8%) cases had Hodgkin lymphoma, 2 (2.2%) cases had Non Hodgkin lymphoma (NHL) and 1 (1.1%) person had burkitt's lymphoma. In addition, 1 (1.1%) patient had both ALL and NHL simultaneously. The mean age of cancer affliction was 6.33 years old.

 The highest incidence of fungal infections was in 2-year-old patients with prevalence of 11, whereas the lowest incidence was in 19-yaer-old patients with prevalence of 1. The minimum age of the patients afflicted by fungal infections was 1 and the maximum age was 19 years old and a mean age 7.95 years old.

 Candidiasis was the most frequent fungal infection among the patients with the prevalence of 65 where Aspergillosis and mucormycosis had the prevalence of 15 and 10, respectively. Some children had two types of fungal infections simultaneously.

 64 patients were infected with oral infections (73.6%) including 59 cases (92.1%) of oral candidiasis and 5 cases (7.9%) of hard palate mucormycosis. The rest of patients were afflicted with infections of other parts of the body (26.4%): 11 patients had upper respiratory infections (12.6%), 10 patients had pneumonia (11.49%), 4 patients had external otitis (4.6%) and 1 patient had dermatitis (1.1%).

 46 patients (52.8%) were in the induction phase of chemotherapy when they were infected with fungal infections: 35 patients (40.4%) in the maintenance phase and 6 patients (6.8%) in the relapse phase. 67 patients (77%) had completely recovered, and unfortunately 20 patients (23%) had died which included 7 of 65 patients afflicted by candidiasis (10.7%), 5 of 10 patients afflicted by mucormycosis (50%) and 8 of 15 patients afflicted by aspergillus (53.3%) while they were receiving treatments.

 There was a significant relationship between age of affliction and incidence of fungal diseases (p <0.0001). In other words, incidence of fungal infection shows a decline in older patients. There was a significant relationship between age and species of microorganisms ( =0.947): all aspergillosis cases occurred at ages of 4-8, whereas candidiasis and mucormycosis were seen at any age. There was no relationship between age and site of infections ( =0.307). 

 There was no significant relationship between gender and age of affliction with fungal diseases and species of microorganism ( = 0.548, p =0.424 respectively). There was a significant relationship between gender and site of infections (p =0.029), where 36% of the girls and 24% of the boys had non-oral infections. In other words, girls with hematologic cancers are more likely to be afflicted by fungal infections in other sites of the body. 

 There was a significant relationship between age of affliction with cancer and age of affliction with fungal infections (p <0.0001) where 88% of the patients were afflicted with fungal infections one year after cancer and 100% of the patients were afflicted after five years. There were no relationships between age of affliction with cancer, species of microorganism and site of infections ( =0.072 and = 0.207 respectively). There were no relationships between age of affliction with fungal infections and species of microorganism, site of infections and type of hematologic cancer ( =0.129, =0.263 and =0.329 respectively).

 There were significant relationships between species of microorganism and site of fungal infections (p <0.0001) and result of the treatment (recovery or dead) (p <0.0001 both). Candidiasis often has oral manifestations, aspergillosis has lower respiratory manifestations and mucormycosis has upper respiratory manifestations. Furthermore, mortality rate of mucormycosis and asergillosis is higher than candidiasis. There was no relationship between the species of microorganism and type of hematologic cancer (p =0.634).

 There was no relationship between site of fungal infections and type of hematologic cancer (p =0.960). There was a significant relationship between site of infections and result of treatment (recovery or dead) (p <0.0001) where patients with oral infections had a better prognosis compared to those with non-oral fungal infections.

**Table I:** Comparative table for Prevalence of difference types of hematologic malignancies among children

	Current study	Previous studies
ALL	72.5%	78%
AML	17.3%	16%
Hodgkin Lymphoma	5.8%	7.1%
NHL	2.2%	6.5%
Burkitt Lymphoma	1.1%	--
ALL with NHL	1.1%	--

ALL: Acute Lymphoblastic Lymphoma, AML: Acute Myeloid Lymphoma, NHL: Non Hodgkin Lymphoma.

**Table II:** Comparative table for Prevalence of fungal infections among children with hematologic malignancies

	Current study	Previous studies
Candidiasis	74.7%	69%
Aspergillusis	17.2%	8%
mucormycosis	11.5%	15%

**Table III:** Comparative table for Mortality rate of children with hematologic malignancies due to fungal infections

Previous studies	Current study	
15%	10.7%	Candidiasis
62.5%	50%	Aspergillusis
65%	53.3%	mucormycosis

**Table IV:** Comparative table for Prevalence of difference sites of fungal infections in children with hematologic malignancies. URS: Upper Respiratory System

**Previous studies** **(rate)**	**Current study** **(rate)**	**Current study** **(Prevalence)**	**Sites of infection**	
**84.2%**	92.3%	60 patients	Oral	Candidiasis
**--**	1.5%	1	URS	
**--**	4.6%	3	External Otitis	
**--**	1.5%	1	Dermatitis	
**56%**	66.6%	10	Pneumonia	Aspergillusis
**--**	33.3%	5	URS	
**--**	40%	4	Oral	Mucormycosis
**--**	50%	5	URS	
**--**	10%	1	External Otitis	

**Table V:** Comparative table for ratios of hematologic malignancies between girls and boys

**Previous studies**	**Current study**	
**2/3**	32/ 55	

**Table VI:** Comparative table for ratios of hematologic malignancies between girls and boys

**No relationship**	$r^{2}$ **=0.129**	**relationships between the ages of affliction with fungal infections and species of microorganism**	**Significant relationship**	***p*** ** <0.0001**	**relationship between the ages and the age of affliction with fungal diseases**
**No relationship**	$r^{2}$=0.263	relationships between the ages of affliction with fungal infections and the site of infections	Significant relationship	$r^{2}$=0.947	relationship between the ages and the species of microorganisms
**N0 relationship**	$r^{2}$=0.329	relationships between the ages of affliction with fungal infections and the type of hematologic cancer	No relationship	$r^{2}$=0.307	relationship between the ages and the site of infections
**Significant relationship**	*p* <0.0001	relationships between the species of microorganism and the site of fungal infections	No relationship	$r^{2}$= 0.548	relationship between the genders and the age of affliction with fungal diseases
**Significant relationship**	*p* <0.0001	relationships between the species of microorganism and the result of treatment for patients (recovery or dead	No relationship	*p*=0.424	relationship between the genders and species of microorganism
**No relationship**	*p*=0.634	relationship between the species of microorganism and the type of hematologic cancer	Significant relationship	*p*=0.029$r^{2}$	relationship between the genders and the site of infections
**No relationship**	*p*=0.960	relationship between the site of fungal infections and the type the hematologic cancers	Significant relationship	*p* <0.0001	relationship between the age of affliction with the cancer and the age of affliction with the fungal infections
**Significant relationship**	*p* <0.0001	relationship between the site of infections and the result of treatment for patients (recovery or dead)	No relationship	$r^{2}$=0.072	relationships between the age of affliction with the cancer and species of microorganism
			No relationship	$r^{2}$= 0.207	relationships between the age of affliction with the cancer and site of infections

**Figure 1:**
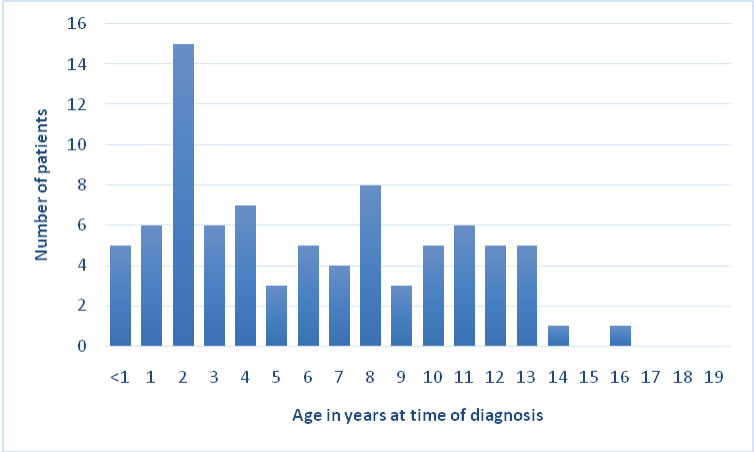
Prevalence of hematologic cancer among children with fungal infections according to current study (72.5% of patients were afflicted by Acute Lymphoblastic Lymphoma)

**Figure 2:**
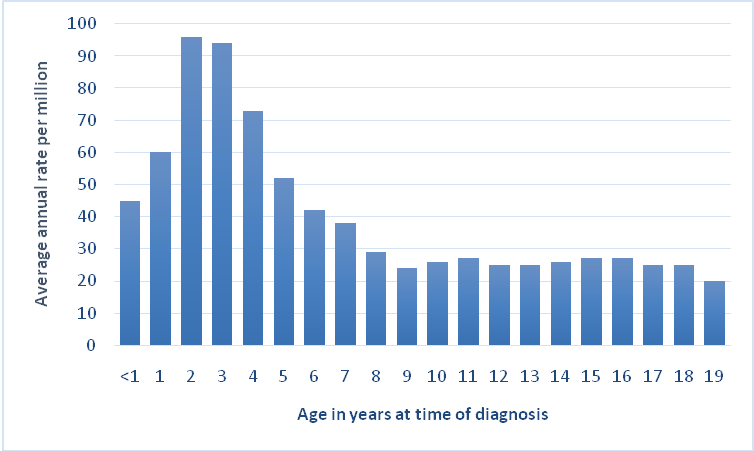
Prevalence of leukemia among of children according to National Cancer Institute (the most common leukemia is Acute Lymphoblastic Lymphoma)

## Discussion

Malignancies have pernicious effects on the immune system which is responsible for detection and elimination of opportunist pathogens. Local and systemic fungal infections are common in patients receiving chemotherapies. On the other hand, prevalence of invasive fungal infections has increased in autopsy of malignant cases. According to retrospective studies, reported prevalence of fungal infections in autopsy between 1919 and 1936 was 0.1 %; however, this number has increased to 1.5% between 1937 and 1941 [10]. Prevalence of invasive fungal infections has demonstrated a growth since 1948, especially in leukemia and lymphoma cases, reaching 20% between 1954 and 1955 [11]. This accelerating rate between 1945 and 1952 is associated with prescriptions of newer cancer drugs such as prednisoloen, mercaptopurine and aminopetrine [10]. Using antibiotics drugs, such as penicillin, tetracycline and streptomycin along with cancer drugs increases longevity of leukemic and lymphatic cases, but increases the chance of invasive fungal colonization and infections [3]. In a study on 8124 bodies autopsied in Germany between 1992 and 1978, the highest prevalence of invasive fungal infections was among acute leukemic cases (31.3%), followed by HIV cases (19.1%), receivers of transplant organs (14.8%), Lymphoma (12.9%) and solid tumors (1.6%) [3].

Candida species are the most common agent of invasive fungal infections in malignant cases in the USA (66%), Aspergillus species accounted for 34% and cryptococcus only in 2% of patients. However, in Germany, according to a study between 1978 and 1992, prevalence of candidiasis decreased from 77.3% to 24.6%, and the rate of aspergillus infections increased from 22.7% to 66.7% [3]. The falling statistics of candidiasis in Germany can be related to timely diagnosis and treatments of candidiasis before the patients' expiration. In addition, diagnosing invasive infections caused by aspergillus species is too complicated before patient's death, mostly detectable in autopsy. Invasive aspergillus is more common among neoplastic patients than non-neoplastic ones, for instance the solid organ transplantations (96.1% cases versus 3.8%) [5]. 

According to global statistics, 31% of malignancies among children under 15 years old and 25% of cancers in patients under 20 years old are the same type of hematologic malignancies. Hematologic malignancy is the most common type of cancer among children [12]. About 3250 children are afflicted with leukemia in the USA every year. 17% of these children are neonates (under 1 year old) and 46% of them are between 2 and 3 years old and the rate decreases at age of 19. In other words, hematologic malignancies have the highest prevalence among children under 1 year old, the rate starts to decrease at age of 10, and the rate shows an accelerating trend until the age of 19. The peak of hematologic cancer rate among children under one year old is higher than that of the other patients. In current study, the age of affliction by hematologic cancers matched global statistics up to age of 10, however, rate showed descending slope as opposed to an accelerating one, which was due to a small sample size. 

 According to the national association of cancer for North America, there are 2 oncologic girl cases versus 3 boy cases, in other words, prevalence of boys is 1.5 times more than that of girls. These data match the results of the current study [12]. 

According to national association, 78% of hematologic cancer cases are ALL, 16% are AML and only 6% of the cases are an uncommon leukemia. Furthermore, according to this statistics, lower prevalent leukemias are the Hodgkin Lymphoma with 7.1% prevalence and NHL with 6.5% prevalence. The results of this study do not exactly match global statistics, but they have many similarities. 

In many studies, the fungal infection in leukemic patients who had suppressed immune system due to chemotherapy is candidiasis albicans. Inasmuch as early diagnosis of cadidiasis in Germany resulted in reduction of cadidiasis rate from 77.3% to 24.6%, we can dramatically decrease mortality due to fungal infections in leukemic patients by improving the diagnostic and screening methods. In addition, the mucormycosis had a lower prevalent in this study which matches global statistics. 

The mean age of affliction by fungal infections was 7.95 years old, which shows most patients were afflicted by the fungal infections due to an immunocompromised system in the induction phase of chemotherapy. During the four-week treatment period of the induction phase in a hospital, many types of anti leukemic drugs are used, making these children susceptible to several infections. After induction phase treatment, 95% of bone marrow samples do not have leukemic cells, but this temporary recovery is accompanied by a severe immunocompromised system. During the maintenance phase, treatments are continued to eradicate other residual leukemic cells which helps with cancer regression. The maintenance phase can take many years during which patients are cared for at home where the risk of infections is higher since most fungal spores are spread in the environment, such as the air, soil, furniture, even toys, etc.

 The species of aspergillus are the most perilous pathogens in hematologic malignancy children, followed by the species of zygomycota. Despite the fact that candidiasis is the most prevalent fungal infection among the children with hematologic malignancy, it is not the main mortality factor among them.

## Conclusion:

According to this retrospective study, timely diagnosis and treatment of fungal diseases are critical in children with hematologic cancer, and dramatically decrease the mortality rate in these children.
